# Impact of Rapid Molecular Multiplex Gastrointestinal Pathogen Testing in Management of Children during a *Shigella* Outbreak

**DOI:** 10.1128/jcm.01652-22

**Published:** 2023-02-28

**Authors:** N. Kanwar, J. Jackson, T. Bardsley, A. Pavia, K. M. Bourzac, K. Holmberg, R. Selvarangan

**Affiliations:** a Children’s Mercy Hospitals and Clinics, Kansas City, Missouri, USA; b University of Missouri, School of Medicine, Kansas City, Missouri, USA; c University of Utah, Salt Lake City, Utah, USA; d bioMérieux, Salt Lake City, Utah, USA; Johns Hopkins University

**Keywords:** BioFire GI panel, shigellosis, *Shigella* detection, outbreak management, IMPACT study, GI panel

## Abstract

Fecal culture for isolation and identification of *Shigella* may take days. The BioFire FilmArray Gastrointestinal (GI) panel (bioMérieux, France) is a PCR-based assay that detects enteric pathogens including *Shigella*/enteroinvasive Escherichia coli (EIEC) in about an hour. The aim of this study was to evaluate the impact of GI panel detection of *Shigella* in a pediatric emergency department (ED) during an outbreak. Stool samples from children with acute gastroenteritis were tested by the GI panel. Test results were either withheld in preintervention (PRE) or reported to clinicians/families in the postintervention (POST) period. The impact of the GI panel testing on patient management and outcomes was measured. *Shigella/*EIEC was identified by the GI panel in the PRE (*n* = 30) and POST (*n* = 21) phase. The GI panel detected more *Shigella* infections than did culture; six of 31 (19.4%) *Shigella* GI panel-positive patients who also had stool cultures were missed by culture. Azithromycin therapy was prescribed for 20% of subjects in the PRE phase and 71.4% of subjects in the POST phase (*P* < 0.001). Time from the clinical encounter until starting azithromycin therapy was shorter in the POST phase (*n* = 9), 8.25 h (range, 6.37 to 52.37 h), than in the PRE phase (*n* = 1), 72 h. Six subjects in the PRE phase visited additional providers compared with one in the POST phase. Prompt diagnosis of shigellosis with the GI panel may provide the opportunity for prompt antimicrobial therapy and avoid additional visits to providers due to early definitive diagnosis. Prompt diagnosis of *Shigella* at an ED visit may optimize patient management and reduce transmission.

## INTRODUCTION

The global incidence of diarrheal diseases has been estimated to be approximately 4.48 billion in the year 2016 (1). Approximately 269.2 million diarrheal episodes in the year 2016 have been attributed to *Shigella* alone with 74.8 million episodes in children less than 5 years (1); *Shigella* is the third most common enteric disease in the United States (2). *Shigella* was responsible for an estimated 75,000 deaths among children under 5 years of age globally in 2016 (3). Shigellosis can range from asymptomatic infection to life-threatening conditions including hemolytic-uremic syndrome, arthralgia, toxic megacolon, and central nervous system disorders (4). Fecal culture for *Shigella* detection is the conventionally accepted gold standard. Culture may take several days for *Shigella* isolation and has limited sensitivity. Culture-independent assays including PCR have the potential to improve patient care with rapid diagnosis and improved sensitivity and specificity (5, 6). The BioFire FilmArray Gastrointestinal (GI) panel (bioMérieux, France) is a PCR-based assay that detects 22 different enteric pathogens including *Shigella* in about an hour. The aim of this study was to evaluate the impact of GI panel detection of *Shigella* in a pediatric emergency department (ED) during an outbreak.

Our hypothesis was that rapid molecular testing and diagnosis of *Shigella* in an outpatient setting are more likely to result in appropriate therapy and reduce repeat health care encounters than is culture.

## MATERIALS AND METHODS

### Study design.

**FIG 1:**
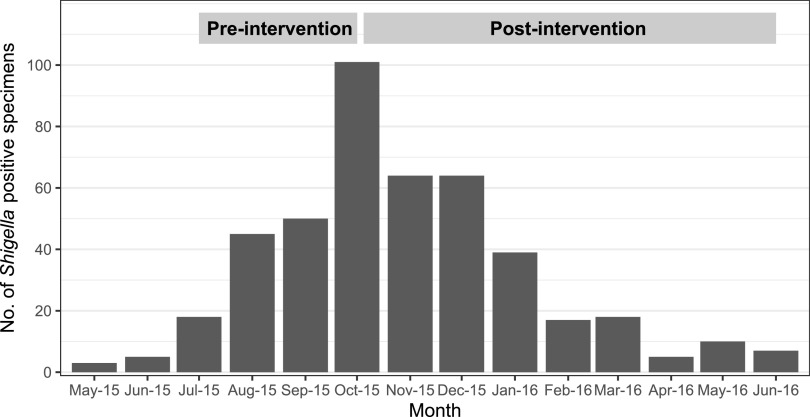
*Shigella* outbreak, Kansas City, MO, 2015 to 2016. Number of *Shigella*-positive specimens from routine standard of care testing (i.e., culture).

This GI IMPACT study was a prospective multicenter study evaluating the impact of implementation of the GI panel on patient management and health outcomes. During the course of the study, one of the five sites (Kansas City, MO) experienced a community-wide *Shigella* outbreak during 2015 to 2016 (Fig. 1) (7, 8). This paper focuses on evaluating the impact of implementation of the GI panel during an outbreak at a single site. The study protocol was approved by the institutional review board. This study was designed to study health outcomes of pediatric subjects presenting to emergency departments with GI illness at hospitals before (PRE phase) and after (POST phase) introduction of the GI panel (i.e., the intervention) amid a *Shigella* outbreak. During the preintervention period, GI panel testing was performed at a central laboratory and results were not reported to the clinicians or patients. During the intervention period, all enrolled subjects received a GI panel test in “real time” at no charge as part of their health care visit; results were included in the subject’s medical records. The GI panel was used in accordance with the manufacturer’s package insert using only preserved stool samples in Cary-Blair transport medium. Reflex culture was performed on any specimen for which the GI panel detected a reportable organism including *Shigella*. Physicians ordered standard of care (SOC) culture assay at their discretion during both phases of the study. Confirmatory identification for *Shigella* from culture was performed by the Vitek 2 Gram-negative card (bioMérieux, France). A stool specimen was requested from all subjects enrolled in the study, regardless of clinician test requests, for both phases. In the preintervention phase, if subjects failed to submit the stool specimen, they were still eligible to stay in the study if they completed the follow-up questionnaire. However, in the postintervention phase, only subjects who submitted the specimens and completed the follow-up questionnaire were included in the study. The instruction sheet and home collection kit were provided along with options for utilizing either the courier services provided through the study or a stool drop-off option at the hospital. IMPACT study analysis including patient outcomes was assessed in pre- and postintervention phases via structured questionnaire at the time of enrollment, medical record review, and follow-up phone interview after 7 to 10 days of subject enrollment.

Male and female children <18 years of age with acute gastroenteritis symptoms for less than 14 days were eligible for inclusion in the study. Subjects were required to provide stool specimens within 48 h of enrollment and provided informed consent/assent, as appropriate. Subjects who had previously been enrolled in the study or subjects with another household member who had been enrolled within the previous 28 days were excluded.

### Data analysis.

Patient management and outcomes were compared between the two phases of this study. Endpoints of the study included (i) detection of shigellosis in children before and after the implementation of the GI panel, (ii) clinical and epidemiologic characteristics of GI infections in pre- and postintervention phases, (iii) impact variables including additional visits to providers, and (iv) rate of azithromycin treatment (empirical versus targeted) and time to treatment. Categorical variables were compared using the Fisher exact test, and continuous variables were compared using the nonparametric Mann-Whitney test. The website http://vassarstats.net/ was utilized for all data analysis.

## RESULTS

**FIG 2:**
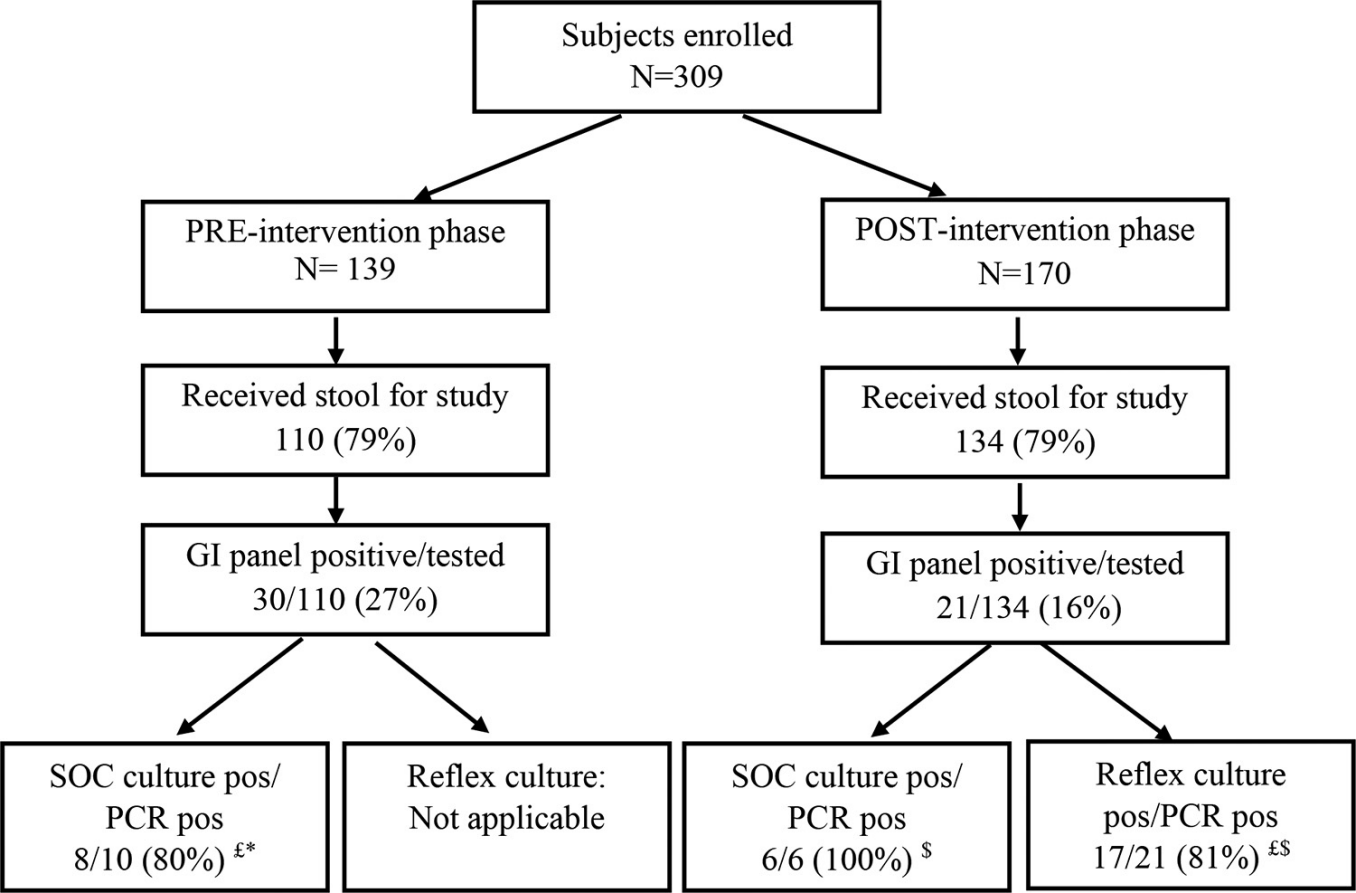
Overall number of subjects in the PRE and POST phases at the Kansas City, MO, site. *, PRE phase: SOC culture performed on 17/110 (15.5%) enrolled subjects. The GI panel detected 10 while SOC culture detected only 8. $, POST phase: SOC culture performed on 20/134 (14.9%) subjects. The GI panel detected 21 while culture detected 17. £, six of the 31 *Shigella* GI panel-positive samples that received culture assay did not grow *Shigella*. pos, positive.

A total of 309 subjects (PRE phase, 139, and POST phase, 170) were enrolled in Kansas City, of which approximately 79% (*n* = 244) of the subjects submitted stool samples. All subjects were enrolled during the *Shigella* outbreak from May 2015 to June 2016 in the Kansas City area (Fig. 1). Twenty-one percent (51/244) of the stool specimens submitted were positive for *Shigella* (Fig. 2 and Table 1). Approximately 41% (*n* = 21) of stool specimens that were positive for *Shigella* had coinfections, with the majority (81%) of coinfections occurring with enteropathogenic Escherichia coli (EPEC; *n* = 10) and/or enteroaggregative E. coli (EAEC; *n* = 7). Physicians ordered stool cultures for only 17 of 139 children (12%) with diarrhea in the ED in the PRE phase. Sixteen of these 17 subjects submitted stool for the study during the PRE period. Ten of the 16 subjects had *Shigella* detected on the GI panel (*Shigella* GI panel positive); SOC culture grew *Shigella* in only eight of these 10 cultures. Similarly, during the POST period, physicians ordered stool cultures for only 20 of 170 children (12%); six of the 20 subjects were *Shigella* GI panel positives; all six samples grew *Shigella* on SOC culture as well. However, in the POST phase, the reflex culture was performed on 27 subjects. Of these 27 subjects, 21 subjects were *Shigella* GI panel positives; only 17 of 21 samples (81%) grew *Shigella* on reflex culture. Overall, all *Shigella* culture-positive samples were detected on the GI panel, and of the 51 *Shigella* GI panel-positive subjects, 31 received culture assay through SOC/reflex culture throughout the study; culture assay missed 6 of the 31 samples (19.4%) that were *Shigella* GI panel positives (Fig. 2). There were no significant differences between patients who were *Shigella* positive by both culture and PCR (*n* = 23) and those positive by only the GI panel (*n* = 6) (see Table S1 in the supplemental material). These results confirm the higher sensitivity of the GI panel than of SOC culture for the detection of *Shigella*. Figure 2 has a detailed description of subject distribution and BioFire GI panel and culture assay results in both phases.

**TABLE 1:** *Shigella*-positive patient demographics and clinical symptoms

Characteristic	PRE (*n* = 30)	POST (*n* = 21)	*P* value
Sex, no.^*a*^	M, 14; F, 16	M, 9; F, 12	1.0
Median age, mo (range)	46 (6–168)	70 (16–180)	0.02
Diarrhea, no. (%)	27 (90)	21 (100)	0.26
Vomiting, no. (%)	13 (43.3)	13 (61.9)	0.26
Fever, no. (%)	25 (83.3)	11 (52.4)	0.03
Diarrheal characteristics/stool consistency (%)			
Median length, days (range)	2 (1–7)	2 (1–7)	0.33
Median no. (range)	5.5 (1–20)	5 (1–27)	0.92
Bloody, no. (%)	6 (22.2)	8 (38.1)	0.21
Watery, no. (%)	21 (77.8)	17 (81)	0.52
Mucous, no. (%)	12 (44.4)	7 (33.3)	0.77
Vomit characteristics			
Median length, days (range)	2 (1–5)	2 (1–5)	0.94
Median no. (range)	4 (0–9)	3 (0–8)	0.14

a M, male; F, female.

The patient characteristics and symptoms did not differ significantly for the *Shigella* GI panel-positive subjects between the PRE and POST phases except age (median, 46 versus 70 months, respectively; *P* value, 0.02) and fever (83% versus 52%; *P* value, 0.03). Fever was reported by 71%, and diarrhea was reported as bloody in 28% (Table 1). The age range for *Shigella*-positive subjects was 6 to 180 months with approximately equal gender distribution. Diarrheal characteristics in terms of stool consistency, number, and length did not significantly differ between the two groups. Similar observations were made for vomiting characteristics as well. Table 1 has a detailed comparison for the two phases.

IMPACT analysis involved comparing the PRE and POST phases for several variables such as visit to additional providers after the initial ED visit, missed work/day care days due to subject’s illness, and the horizontal spread of disease to other family members. Results from completed follow-up questionnaires from *Shigella*-positive subjects revealed that only one subject (5%) in the POST phase had additional visits to a health care provider compared with six subjects (20%) in the PRE phase, although the difference was not clinically significant (*P* = 0.2). Other outcome variables including days of school or day care missed or parental days of work missed were also not significantly different between the two groups (Table 2).

**TABLE 2:** IMPACT variables in the PRE and POST study phases

IMPACT variable	PRE (*n* = 30)	POST (*n* = 19)^*a*^	*P* value
No. of additional health care visits (%)	6 (20) (5 outpatient, 1 ED)	1 (5.3)	0.22
No. of parents who missed workdays (%)	13 (43.3)	10 (52.6)	0.57
Avg no. of days missed by parents (range)	1.8 (1–4)	2.4 (1–5)	0.42
No. of subjects who missed school/day care (%)	22 (73.3)	13 (68.4)	0.75
Avg no. of days missed by subjects (range)	2.8 (1–8)	2.8 (1–5)	0.94
Disease spread among family members, no. positive/total no. (%)	8/133 (5.3)	4/86 (4.7)	1.0

a Follow-up interview was completed by 19 of the 21 *Shigella*-positive subjects.

Appropriate antibiotic treatment, however, was significantly different. Overall, azithromycin treatment was administered to 71.4% of the patients with *Shigella* in the POST phase compared with 20% in the PRE phase (*P* < 0.001). Five of six patients with shigellosis who received azithromycin in the PRE period were treated empirically; that is, the physician did not have laboratory evidence of *Shigella*. Targeted treatment after the GI panel result was reviewed by the medical provider in the POST phase was observed in 42.9% of the subjects, while only 3.3% of the subjects in the PRE phase (*P* < 0.001) received treatment in the absence of the GI panel test result. There were five patients who received an antibiotic other than azithromycin—three in the PRE group (trimethoprim-sulfamethoxazole [*n* = 2] and amoxicillin [*n* = 1]) and two in the POST group (ciprofloxacin [*n* = 1] and cefdinir [*n* = 1])—among the *Shigella* GI panel positives. Azithromycin was prescribed to only one of all the patients who were *Shigella* GI panel negative; this patient was positive for Campylobacter (Table S2). There was unnecessary antibiotic usage observed in both phases among patients for whom the *Shigella* GI panel was negative; however, there was no significant difference found for unnecessary antibiotic usage between the non-*Shigella* groups in PRE (*n* = 7, 9%) and POST (*n* = 11, 10%) phases (*P* value = 1.0). Median time to targeted treatment was 8.3 h (range, 6.4 to 52.4 h) in the POST phase for nine subjects (42.9%) compared with 72.3 h for only one subject in the PRE phase, where treatment was changed after culture results were available (Table 3). The decision to not treat subjects with azithromycin seemed to be influenced by the disease severity in the POST phase (Table 4). There were less severe symptoms and fewer coinfections in the azithromycin-nontreated group in the POST phase than in that group in the PRE phase. Similarly, time to ED visit after symptom onset was greater in the nontreated group (Table 4).

**TABLE 3:** Treatment in the PRE and POST study phases^*a*^

Characteristic	PRE (*n* = 30)	POST (*n* = 21)	*P* value
Treatment, no. (%)			
Azithromycin treatment	6 (20)	15 (71.4)	<0.001
Empirical treatment	5 (16.7)	6 (28.6)	0.49
Targeted treatment (culture, PRE; FilmArray, POST)	1 (3.3)	9 (42.9)	<0.001
Time to Rx-ALL (h), median (range)	2.31 (1.21–72.32)	6.45 (1.14–52.37)	0.41
Time to targeted Rx (h) (range) (*n* = 1, PRE; *n* = 9, POST)	72.32	8.25 (6.37–52.37)	

a Rx, azithromycin treatment; Rx-ALL, all subjects that received Emperic/targeted azithromycin treatment.

**TABLE 4:** Factors influencing treatment in the PRE and POST study phases

Factor	PRE (*n* = 30)		POST (*n* = 21)	
	Treated with azithromycin (*n* = 6)	Not treated with azithromycin (*n* = 24)	Treated with azithromycin (*n* = 15)	Not treated with azithromycin (*n* = 6)
Time—onset of symptom to ED visit, median (range) days	1.5 (0–2)	1 (0–6)	2 (0–6)	2 (1–4)
No. of diarrheal episodes, median (range)	7 (5–13)	5.5 (1–20)	5.5 (1–27)	3.5 (1–13)
Bloody diarrhea, no.	3	3	7	1
Oral rehydration, no.	1	10	7	6
Coinfections, no.^*a*^	5, *Shigella*; 1, multiple pathogens	10, *Shigella*; 14, multiple pathogens	12, *Shigella*; 3, multiple pathogens	3, *Shigella*; 3, multiple pathogens

a Among the coinfections, EPEC was the most common pathogen as detected by the GI panel.

## DISCUSSION

We observed the impact of multiplex PCR stool testing on *Shigella* detection and treatment in a prospective study that occurred during a community-wide *Shigella* outbreak in Kansas City, MO. Despite widespread publicity about the increase in pediatric *Shigella* infections, only 37 subjects (12%) also received physician-ordered stool cultures. *Shigella* was isolated from 15 samples (41%). All culture-positive samples were also positive with the GI panel (14/14; 100%); one *Shigella* culture-positive subject did not submit stool for GI panel testing. However, of these 51 *Shigella* GI panel-positive subjects, 31 received culture assay through SOC/reflex culture throughout the study. Culture assay missed six of the 31 samples (19.4%) that were *Shigella* GI panel positive. The GI panel detected approximately 20% more *Shigella*-positive specimens than did culture requested as the standard of care test in both phases and the reflex culture after positive *Shigella* detection by GI panel in the POST phase. The clinical utilization of this molecular assay in the real world will be dictated by SOC testing orders (SOC culture orders were requested for only 12% of the patients in our study). However, one of the main factors that may influence provider behavior is the faster turnaround time for the assay; it is possible that they may place more SOC orders when quick molecular assay options are available and will wait to start targeted therapy as opposed to empirical antibiotic treatment.

The BioFire GI panel does not distinguish between *Shigella* and enteroinvasive E. coli (EIEC), and samples positive for *Shigella*/EIEC by the GI panel were not further confirmed by independent PCR assay and sequencing. However, the results were likely to represent *Shigella* given the timing of the specimen collection during the outbreak. Previous studies comparing molecular PCR-based assays with culture for *Shigella* detection have similar findings of higher detection rates for the molecular biology-based assays (9–13). The more sensitive molecular assays like the GI panel requiring minimum technical expertise can aid in increased diagnostic accuracy, resulting in prompt clinical management, and can also be utilized as a surveillance tool for more accurate prevalence estimates.

We found no statistically significant differences in clinical outcomes of impact on the family between the two periods at follow-up. However, the number of patient repeat visits with medical providers was higher in the PRE phase (20%), compared with one in the POST phase (*P* = 0.2).

The major significant impact was observed on antibiotic treatment. Azithromycin treatment was administered to significantly more subjects in the POST phase (71.4%) than the PRE phase (20%) (*P* < 0.001). We also noted an increase in targeted treatment and a shorter time to targeted treatment. Another study that evaluated the impact of the GI panel on patient management reported that the patient group where GI panel results were provided had significantly fewer days on antibiotics, fewer days to discharge, fewer imaging studies (abdominal/pelvic) ordered, and overall decreased health care cost (14). A cost-benefit analysis study performed in London, United Kingdom, for another GI multiplex panel (Luminex xTAG gastrointestinal pathogen panel) similarly found faster turnaround time to diagnosis and greater sensitivity than in the conventional method (15).

Molecular testing was performed using only stool specimens preserved in Cary-Blair medium, which could have limited study participation. Rectal swab samples may be easier to collect at point-of-care and provide faster test results, but the suitability of this method for molecular testing needs further investigation. Additionally, potential benefit with result availability to the patients in the POST phase could have led to enrollment bias. Reflex culture was performed only when a reportable bacterial pathogen (e.g., *Shigella*, Salmonella, and E. coli) was detected by the GI panel to confirm molecular assay results. This potentially limits the assessment of the molecular method.

Appropriate treatment of *Shigella* infection can shorten the duration of symptoms, decrease shedding, and potentially decrease transmission, especially during outbreaks (4, 16). Despite early recognition of the outbreak and educational efforts aimed at treating children with symptoms of acute bacterial gastroenteritis resembling shigellosis, only five (17%) of 30 children with *Shigella* detected by PCR in the PRE phase were given appropriate empirical therapy. Many of the children in the POST group would have failed to receive appropriate antibiotic treatment if the multiplex PCR test result had not been available quickly. Our study provides evidence that rapid multiplex molecular testing for GI pathogens in outpatients during an outbreak helps to identify patients, improves targeted antimicrobial therapy, and potentially decreases overall health care cost due to fewer repeat visits.
