# Supplementary material for: Impact of Rapid Molecular Multiplex Gastrointestinal Pathogen Testing in Management of Children during a Shigella Outbreak
Source: J Clin Microbiol. 2023 Feb 28;61(3):e01652-22. doi: 10.1128/jcm.01652-22 (PMC10035298; doi:10.1128/jcm.01652-22)
Supplement: Supplemental file 1 — Supplemental material. Download jcm.01652-22-s0001.pdf, PDF file, 0.06 MB [file jcm.01652-22-s0001.pdf]

Supplemental Table 1.: ***Shigella*** **positive patient demographics and clinical symptoms**

	Shigella **GI Panel positive and culture negative (n=6)**	***Shigella*** **GI Panel and culture positive (n= 23)**	***P*** **value**
**Sex**	M:2<br>F: 4	M: 9<br>F: 14	1.00
**Median Age in months (range)**	23 (10-180)	70 (14-175)	0.12
**Diarrhea (%)**	6 (100%)	23 (100%)	1.00
**Vomiting (%)**	3 (50%)	10 (43.5%)	1.00
**Fever (%)**	2 (33.3%)	12 (52.2%)	0.65
**Diarrheal characteristics/ Stool consistency (%)**	Median length: 4.5 (1-7) days<br>Median no: 6 (2-15)<br>Bloody: 1 (16.7%)<br>Watery: 4 (66.7%)<br>Mucous: 3 (50%)	Median length: 2 (1-5) days<br>Median no: 5 (1-27)<br>Bloody: 11 (47.8%)<br>Watery: 17 (73.9%)<br>Mucous: 10 (43.5%)	0.14<br><br>0.67<br>0.35<br>1.00<br>1.00
**Vomit Characteristics**	Median length: 2 (1-5) days<br>Median no.: 3 (1-8)	Median length: 2(1-3) days<br>Median no.: 3 (0-4)	.

Supplemental Table 2.: IMPACT variables in the PRE and POST study phase by *Shigella* detection

IMPACT variables	PRE		*P*-value	POST		*P*-value	Overall Shigella (n=49)	Overall non-*Shigella* (n=186)	*P*-value
	Shigella (30)	Non-Shigella (76)[a]		Shigella (19)[b]	Non-Shigella (110)[c]				
**Patients with Additional healthcare visits**	6 (20%)	8 (10.5%)	0.21	1 (5.3%)	24 (21.8%)	0.12	7 (14.3%)	32 (17.2%)	0.67
**No. of parents that missed workdays**	13 (43.3%)	33 (43.4%)	1.00	10 (52.6%)	39 (35.5%)	0.20	23 (46.9%)	72 (38.7%)	0.33
**Average no. of days missed by parents**	1.8 (1-4)	2.4 (1-7)	0.81	2.4 (1-5)	2.5 (1-7)	0.23	2.1 (1-5)	2.4 (1-7)	0.47
**No. of subjects that missed school/day care**	22 (73.3%)	30 (39.5%)	0.002	13 (68.4%)	42 (38.2%)	0.02	35 (71.4%)	72 (38.7%)	<0.001
**Average no. of days missed by subjects**	2.8 (1-8)	2.3 (1-6)	0.004	2.8 (1-5)	3.0 (1-10)	0.03	2.8 (1-8)	2.7 (1-10)	<0.001
**Disease spread among family members**	8 /133 (6.0%)	23/321 (7.2%)	0.69	4 / 86 (4.7%)	46/475 (9.7%)	0.15	12/219 (5.5%)	69/796 (8.7%)	0.12
**Azithromycin treatment**	5 (16.7%)	0 (0%)	0.001	9 (47.4%)	1* (0.9%)	<0.001	14 (28.6%)	1 (0.5%)	<0.001

[a] Follow up interview was completed by 76 of the 80 non-*Shigella* subjects in the PRE phase of the study

[b] Follow up interview was completed by 19 of the 21 *Shigella* positive subjects in the POST phase of the study

[c] Follow up interview was completed by 110 of the 113 non-*Shigella* subjects in the POST phase of the study

*Patient positive for campylobacter
